# Biomechanical Characterization of Human Soft Tissues Using Indentation and Tensile Testing

**DOI:** 10.3791/54872

**Published:** 2016-12-13

**Authors:** Michelle Griffin, Yaami Premakumar, Alexander Seifalian, Peter Edward Butler, Matthew Szarko

**Affiliations:** ^1^Division of Surgery & Interventional Science, University College London (UCL); ^2^Anatomy Department, St Georges University; ^3^Plastic & Reconstructive Surgery Department, Royal Free Hospital

**Keywords:** Bioengineering, Issue 118, compression, tensile, indentation, cartilage, skin, biomechanics, biomaterial, regenerative medicine, tissue engineering

## Abstract

Regenerative medicine aims to engineer materials to replace or restore damaged or diseased organs. The mechanical properties of such materials should mimic the human tissues they are aiming to replace; to provide the required anatomical shape, the materials must be able to sustain the mechanical forces they will experience when implanted at the defect site. Although the mechanical properties of tissue-engineered scaffolds are of great importance, many human tissues that undergo restoration with engineered materials have not been fully biomechanically characterized. Several compressive and tensile protocols are reported for evaluating materials, but with large variability it is difficult to compare results between studies. Further complicating the studies is the often destructive nature of mechanical testing. Whilst an understanding of tissue failure is important, it is also important to have knowledge of the elastic and viscoelastic properties under more physiological loading conditions.

This report aims to provide a minimally destructive protocol to evaluate the compressive and tensile properties of human soft tissues. As examples of this technique, the tensile testing of skin and the compressive testing of cartilage are described. These protocols can also be directly applied to synthetic materials to ensure that the mechanical properties are similar to the native tissue. Protocols to assess the mechanical properties of human native tissue will allow a benchmark by which to create suitable tissue-engineered substitutes.

**Figure:**
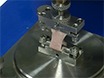


## Introduction

Patients are increasingly waiting for various organ transplantations to treat failing or injured organs. However, with the shortage of suitable donor organs, regenerative medicine is aiming to create alternative solutions for patients with end-stage organ failure. Regenerative medicine aims to meet this clinical need by engineering materials to act as tissue substitutes, including soft tissues, such as cartilage and skin. To create a successful material to restore damaged tissues, the replacement material should mimic the properties of the native tissue it is going to replace^1-2^. Once surgically implanted, the material will need to provide anatomical shape to the tissue defect and thus, the mechanical properties of the material are vital^1^. For example, a material replacing auricular cartilage should have the appropriate mechanical properties to prevent compression by the overlying skin^2^. Similarly, a material to replace nasal cartilage will need to have adequate mechanical properties to prevent collapsing during breathing^3^. However, despite the importance of mechanical properties when manufacturing materials for implantation, little evidence has focused on characterizing the mechanical properties of different human tissues.

Mechanical testing regimes can be used to establish the compressive, tensile, bending, or shear properties of a tissue. Skin is a highly anisotropic, viscoelastic, and nearly incompressible material^4-9^. Commonly excised skin is tested using uniaxial tensile methodologies, where a suitably shaped strip of skin is gripped at both ends and stretched while the load and extension are recorded^4-9^.

Since the major component of all soft tissues is interstitial water, the mechanical response of cartilage is strongly related to the flow of fluid through the tissue^10-11^. Soft tissues such as cartilage have been traditionally tested using compression testing. The methods for testing in compression are quite varied, with confined, unconfined, and indentation being the most prevalent (**Figure 1**). Within confined compression, a cartilage sample is placed in an impervious, fluid-filled well and loaded through a porous plate. Since the well is non-porous, flow though the cartilage is in the vertical direction^12-13^. In unconfined compression, the cartilage is loaded using a non-porous plate onto a non-porous chamber, forcing the fluid flow to be predominantly radial^12-13^. Indentation is the most frequently used method for evaluating the biomechanical properties of cartilage^12-13^. It consists of an indenter, smaller than the surface of the specimen being tested, that is brought down onto the specimen. Indentation has many advantages over other methods of compression, including the fact that indentation can be performed *in situ*, enabling the test to be more physiological (**Figure 1**)^12-13^.

To understand the compressive and tensile properties of a tissue, the Young's elastic modulus is typically calculated by analyzing the linear portion of the stress-strain curve, indicating the elastic resistance to compression or tension, irrespective of specimen size^12^. Both tensile and compressive testing regimes can vary according to the load or deformation applied and the rate of both such parameters. At present, there are many different testing protocols to assess tissue mechanics, which makes it extremely difficult to interpret or compare results from different studies^6-13. ^Furthermore, many mechanical methods currently focus on characterizing the mechanical properties of the tissue by testing the specimen to destruction.We aim to demonstrate an indentation and tensile protocol that provides direct, non-destructive comparison of human soft tissues and tissue-engineered constructs.

We demonstrate a method that limits the mechanical tests to stress yet still obtains a Young's elastic modulus in compression and tension. The sample is stressed either in tension or compression to a certain value, and once the chosen stress value has been reached, the sample is allowed to relax whilst all the data is recorded. This method captures both the viscoelastic and relaxation properties of the tissue within the same test, which can be applied directly to the synthetic material. We have used the indentation protocolto evaluate human soft tissues, including skin and cartilage^14-16^. Cartilage is assessed using indentation testing and skin is evaluated using tension testing^14-16^. Researchers aiming to engineer materials with similar properties to human soft tissues could consider implementing these protocols.

## Protocol

This protocol follows the ethical guidelines of our institution's human research ethical committee guidelines on the use, storage, and disposal of human tissue. Human tissue samples can be excised from cadaveric bodies that have been consented for research purposes with relevant ethical approvals. Samples can also be discarded tissue from consented patients undergoing surgical procedures, with relevant ethical approval.

### 1. Preparation of Skin

1. Prepare specimens by manually dissecting off the adipose tissue and the thin layer of deep dermis using a scalpel blade and forceps. This step is important to ensure consistency between samples^14^.
2. Cut the resulting sheet of split-thickness skin into a standardized sample size (*e.g.,* 1 cm × 5 cm samples). Determine the specimen size based on the dimensions of the testing apparatus. If a tissue-engineered construct is also being tested, the specimen size should be appropriate for the material of interest^14^. Dispose of scalpel blades in the appropriate sharps bins.
3. To enable completion of the mechanical calculations, measure the thickness of the skin being tested using electronic calipers before and after mechanical testing.

### 2. Tensile Testing

NOTE: All materials testing machines should be calibrated according to the manufacturer's guidelines prior to testing.

1. Test skin samples in uniaxial tension using a materials testing machine (**Figure 2A**) at room temperature (22 °C)^14^.
2. Orientate the skin samples in the same direction for all samples (*e.g.,* perpendicular or in-line with Langer Lines (topological lines drawn on a map of the human body and referring to the natural orientation of collagen fibers in the dermis))^14^.
3. Immobilize the sample between two clamps (a commercial jig), one affixed to a 98.07 N load cell and the other to an immovable base plate^14^. The resulting area between the clamps tested in uniaxial tension should be 1 cm x 4 cm (**Figure 2**). NOTE: A commercial jig was utilized to avoid non-uniform gripping and damage to the sample before testing. The sample is fixed to a "finger-tight" tightness.
4. Cover the sample area (after placement in the apparatus) on both sides with petroleum jelly to prevent specimen desiccation.
5. Program the tensile loading and relaxation testing regime into the software as a list of actions, as follows: Zero Load | Zero Position | Find Contact (Tensile loading) | Wait (Relaxation).
6. Start the test with the software program. Load the sample under tension to 29.42 N at 1 mm/s. Use a rate and load that does not cause failure of the skin (*e.g.,* 29.42 N at 1 mm/s).
7. After the 29.42 N-load is reached, allow the tissue to relax for 1.5 h, a time-point at which there is minimal change in relaxation behavior, controlled by the computer software^14^. Note: The displacement is held constant during the relaxation phase, not the load.
8. Calculate elastic and viscoelastic properties as per the analysis section guidelines. The mechanical properties investigated will represent the average properties of the split-thickness skin constituents (epidermis and dermis)^14^. Note: There is no defined tare load, as it is clear from the raw data when deformation is occurring and thus, only these data points are included.

### 3. Preparation of Cartilage

1. Remove the skin and fascia from the cartilage specimen using a scalpel blade and forceps^15^^,^^16^.
2. Divide the cartilage specimens into a standardized sample size (*e.g.,* 1.5-cm blocks) using a scalpel blade and forceps. For all samples, use a semicircular-shaped indenter (**Figure 2B**) that has a diameter and thickness at least 8 times greater than the size of the cartilage sample. This ratio ensures that the indenter is not affected by any edge effects from specimen preparation^15^. Dispose of scalpel blades in the appropriate sharps bins.
3. To enable completion of the mechanical calculations, measure the thickness of the cartilage to be loaded using electronic calipers before and after mechanical testing^15^^,^^16^.

### 4. Compressive Indentation Testing

1. Compress the cartilage samples using a materials testing machine in a hydrated environment at room temperature. Cover the cartilage sample with phosphate-buffered saline (PBS) prior to and during compression testing to ensure that the sample is hydrated. NOTE: PBS does not exactly match the physiological environment, but it allows both the materials and the tissues to be compared equally^15^^,^^16^.
2. Orientate the cartilage sample so the surface is perpendicular to the indenter. This allows the compression to be uniaxial and limits any shear loading^15^.
3. Program the compressive loading and relaxation testing regime into the software as a list of actions, as follows: Zero Load | Zero Position | Find Contact (Compressive loading) | Wait (Relaxation).
4. Start the test using the software program. Load the sample under compression to 2.94 N at 1 mm/s^15^^,^^16^. NOTE: This was determined to be a non-destructive load that is sensitive enough to identify both elastic and viscoelastic properties of cartilage^15^.
5. After the 2.94-N limit is reached, allow the cartilage to relax for 15 min, a time-point at which there is minimal change in relaxation behavior, using the computer software^15^^,^^16^. NOTE: **Figure 2C-D** shows a typical set up for the compression and tensile testing of human tissue specimens. The same protocols can then be applied to synthetic biomaterials to match the biomechanical properties to the native tissue being analyzed. For example, **Figure 2E-F** demonstrates compression and tensile testing of human tissue closely matching a synthetic material's biomechanical properties.

### 5. Calculation of Young's Elastic Modulus for Indentation and Tensile Testing

1. Collect the raw data including time (s), displacement (mm), and load (N) from the materials testing device^14-16^.
2. Calculate the stress (MPa) and strain (%) using the formulas shown in **Figure 3**. NOTE: If a hemispherical indenter was used during compression testing, dividing the force by the cross-sectional area gives the nominal (average) stress, but not the peak stress.
3. Use a linear scatter plot to plot the stress MPa (y-axis) against the strain (x-axis). Determine the linear curve fit. The linear curve fit is equal to y = mx + b with a respective R value. NOTE: All data points are included to achieve a minimum R value >0.98. The m value is the slope, which corresponds to the modulus of stress over strain, indicating compressive resistance or resistance to tension in MPa (*i.e.,* Young's Modulus). If the R value is not >0.98, then the assumption of characterizing linear viscoelastic behavior is invalid.
4. To identify the viscoelastic properties in which fluid flow from exposure to deformation has reached equilibrium, the ratio of stress over time over the last 200 s of mechanical testing and the final stress level at the end of the experiment are calculated. NOTE: With increasing time, the stress level will decrease (relax) as fluid flow reaches equilibrium^17^^,^^18^. A fast stress-relaxation response indicates that it is difficult to maintain high stresses within the sample^17^^,^^18^.

### 6. Relaxation Properties

1. Plot stress in MPa (y-axis) against time in s (x-axis) on a linear scatter plot.
2. Determine a linear curve fit to calculate the rate of relaxation. The linear curve fit is equal to y = mx + b with a respective value of the last 200 s. The m value is the rate of relaxation.
3. Include all data points to obtain a minimum R value >0.98. The final stress (MPa) at 1.5 h for skin and 15 min for cartilage is the final absolute relaxation value.

## Representative Results

**Figures 4 **and** 5** provide examples of data obtained via indentation and tensile testing. **Figure 4** demonstrates typical values obtained after human cartilage indentation testing. **Figure 4A** is an example of a typical strain-versus-stress plot obtained after indentation testing. To obtain the Young's Modulus, all values are included until the line curve fit has a minimum R value of 0.98 (**Figure 4B**). The m value is the indicator of Young's Modulus in MPa; for example, in this data, the cartilage has a modulus of 1.76 MPa. **Figure 4C** shows a typical plot of stress against time to evaluate the relaxation properties of cartilage. The rate of relaxation is calculated from the last 200 s. Similarly, to obtain the rate of relaxation, the m value of a line curve fit in MPa is used. For example, in this data, the cartilage has a rate of relaxation of 8.78 x 10^-6^ MPa/s (**Figure 4D**). The absolute final level of relaxation is the final point of stress in MPa. For example, in this data set, the absolute final level of relaxation would be 0.028 MPa (**Figure 4D**).

**Figure 5** shows how to evaluate the viscoelasticity of skin tissue after tensile testing. The analysis is carried out as per compression testing. **Figure 5A** demonstrates a typical strain-versus-stress plot obtained from the tensile testing protocol. To obtain the Young's Modulus in tension, all values are included until the line curve fit has a minimum R value of 0.98 (**Figure 5B**). The m value is the indicator of Young's Modulus in MPa; for example, in this data, the skin has a modulus of 0.62 MPa. **Figure 5C** shows a typical plot of stress against time to evaluate the relaxation properties of skin. The rate of relaxation is calculated from the last 200 s. Similarly, to obtain the rate of relaxation, the m value of a line curve fit in MPa is used. For example, in this data, the skin has a rate of relaxation of 3.1 x 10^-5^ MPa/s (**Figure 5D**). The absolute final level of relaxation is the final point of stress in MPa. For example, in this data set, the level would be 0.64 MPa (**Figure 5D**). The same analysis can then be utilized to analyze biomaterials under compression and tensile testing to match their biomechanical properties to native tissue.


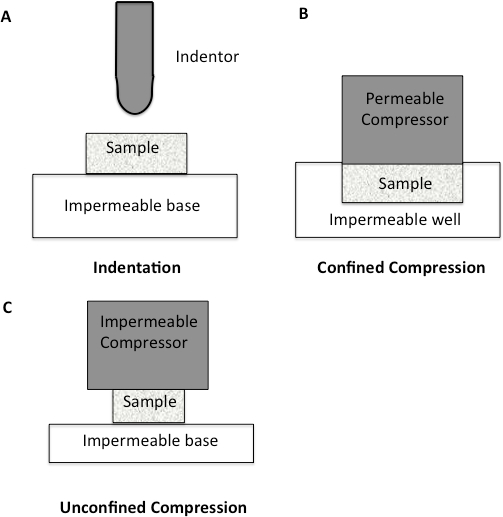
**Figure 1: Schematic diagram to illustrate different compression methodologies. A. **Indentation Testing. A load is applied to a small area of the cartilage using a non-porous indenter. **B. **Confined Compression. The cartilage specimen is placed in an impervious fluid-filled well. The cartilage is then loaded through a porous plate. Since the well is impervious, flow through the cartilage is only in the vertical direction. **C.** Unconfined Compression. The cartilage is loaded using a non-porous plate onto a non-porous chamber, forcing fluid flow to be predominantly radial.


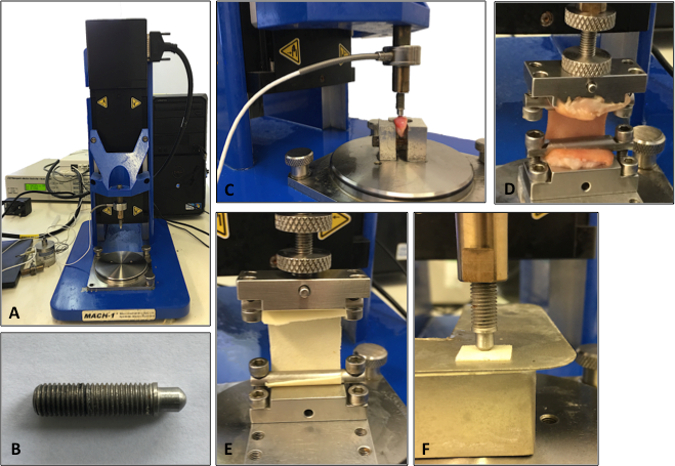
**Figure 2:Set-up of the mechanical testing machine. A.** Illustration of the testing machine. **B**. Illustration of the indenter used for the compression testing analysis. **C.** Cartilage being analyzed using compression indentation testing. **D.** Skin tissue being analyzed under tensile testing. **E.** Tensile testing of a synthetic biomaterial. **F.** Compression testing of a synthetic biomaterial.


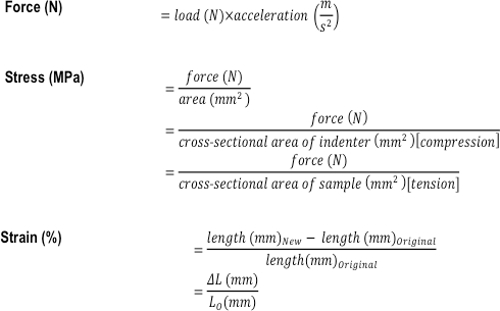
**Figure 3:Formulas used to calculate the compressive and tensile mechanical properties of a tissue or tissue-engineered construct. **The formulas used to calculate force (N), stress (MPa), and strain (%).


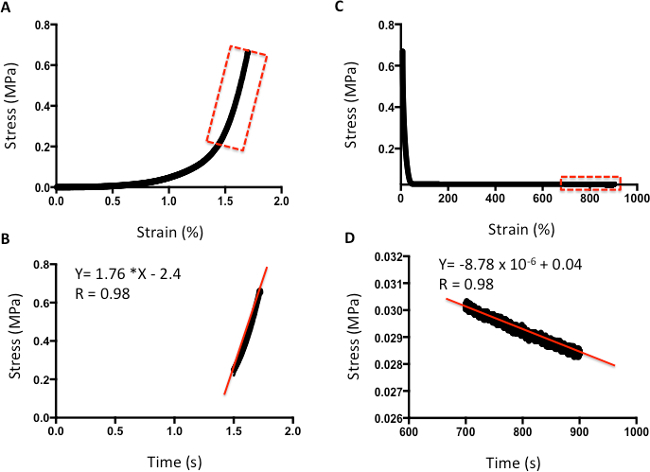
**Figure 4: Example of compression analysis of human cartilage. A.** Stress-versus-strain analysis. **B.** The m value of the line curve fit equation is the Young's Elastic Modulus in MPa. **C.** Stress-versus-time analysis to demonstrate relaxation properties. **D.** The m value of the line curve fit equation indicates the relaxation rate. The final absolute rate is the last point on the graph.


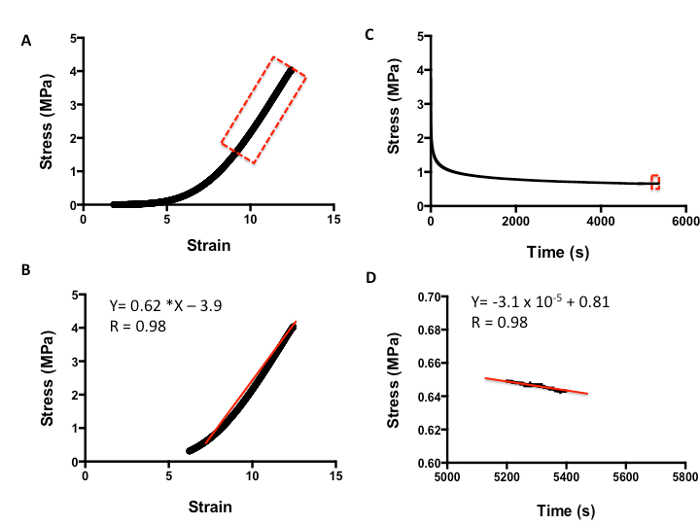
**Figure 5: Example of tensile analysis of human skin. A.** Stress-versus-strain analysis. **B.** The m value of the line curve fit equation is the Young's Elastic Modulus in MPa. **C.** Stress-versus-time analysis to demonstrate relaxation properties. **D.** The m value of the line curve fit equation equates to the relaxation rate. The final absolute rate is the last point on the graph.

## Discussion

Several tensile and indentation protocols have been published to characterize human soft tissues. We have provided another method, which aims to be more diagnostic and non-destructive. The samples undergoing mechanical testing in this protocol are limited by load rather than by displacement, as transducers are more sensitive to load than to displacement. Therefore, reproductions of the experiment can be more precise across tissues and synthetic materials. Using this technique, we have demonstrated a tensile protocol for evaluating skin tissue and an indentation protocol for analyzing cartilage tissue. Both protocols are easy and simple to implement and could be considered for the characterization of human soft tissues and tissue-engineered constructs.

One of the vital steps of the methodology to obtain a stress-relaxation curve suitable for analysis is to ensure that the sample does not slip during testing. Adequate fixation is required, but this must be balanced against causing any stress on the specimens and ensuring that the indenter is perpendicular to the surface to prevent any shear loading. It is critical that the composition as well as size and shape of the tissue are similar between samples. For cartilage, it is vital to use a repeatable dissection protocol and sample dimensions. For skin samples, it is vital to remove all the subcutaneous tissue in order to obtain a repeatable sample. It is also important to ensure that for all samples, the specimen conditions are identical, including hydration, room temperature, and thawing process, if appropriate.

There are some limitations to the protocols presented. Studies have suggested that deformation characteristics of skin and cartilage are dependent upon specimen orientation^13^. Skin was recognized to be anisotropic as far back as the 19^th^ century, with Langer demonstrating in 1861 that the skin has natural lines of tension, referred to as Langer lines^4^. Thus, when characterizing skin samples, it is important to orientate all samples parallel or perpendicular to the Langer Lines to avoid introducing a methodology bias^4^. Cartilage also shows anisotropic properties and contains Hultkrantz lines, which are equivalent to Langer lines, so the cartilage can deform differently according the direction in which it is loaded^12^^,^^19^. Thus, it is important to increase the sample size to allow for the testing of cartilage in different directions. As biomechanical properties of tissue also vary with age and gender, studies should be performed with a representative patient cohort to maintain validity to the clinical setting. Furthermore, some mechanical protocols advocate preconditioning, where the tissue undergoes cyclic loading to ensure that the tissue is in a steady state for subsequent mechanical testing^20^. However, the exact mechanism of preconditioning is unclear and the exact number of cycles needed to produce a consistent and repeatable response varies in different studies^20^. The researcher should consider whether or not to include preconditioning after evaluating the reason for performing the specific biomechanical test^20^.

Skin is a complex, multi-layered material, divided into three main layers: the epidermis, dermis, and hypodermis^4^.The mechanical properties of skin tissue have recently been evaluated using *in vivo* assessments^4^.However, protocols of tensile testing can be utilized to understand the skin biomechanics of excised skin^4^. Such tests can provide information to model stress-strain relationships, since the boundary conditions can be defined^4^. Typically, *in vitro* testing regimes use high strains to characterize the material to failure, whereas *in vivo* systems use low strain ranges^4^. When comparing biomechanical values for excised skin in tension, there is a large variability between different studies, ranging from 2.9-150 MPa^4^. Large differences between subjects are expected due to natural biological variation, but differences in protocol regimes can also compound these natural biological differences. For example, differences in loading rates between protocols will cause variation, as greater loading rates cause less time for the fluid to flow out, resulting in a higher stiffness. The preparation, excision, and handling protocols of the skin tissue will also cause differences in the mechanical properties^4^. This protocol demonstrated for testing skin provides an alternative method for researchers to characterize skin tissue. It provides a few advantages, including the ability to identify the elastic and viscoelastic properties of skin tissue in one mechanical test, allowing for a greater understanding of the skin in a short amount of time. Furthermore, the same test can be applied to tissue-engineered replacements to manufacture constructs with similar biomechanical properties as native skin.

Indentation testing provides an attractive option compared to confined compression testing for understanding the biomechanics of cartilage^21^. Indentation has the ability to preserve the physiological structure of the cartilage and thus provides values that mimic those of a clinical setting. Using indentation, it is also possible to test the cartilage while still attached to the underlying bone. Indentation also allows for physiological testing of cartilage as *in vivo*. When two cartilage surfaces approach each other, the edges surrounding the area of contact "bulge" due to water under the contact area being displaced laterally after compressive deformation occurs^17^^,^^21^. Cartilage indentation must be conducted with an indenter with a smaller radius than the cartilage sample to allow for similar bulging. The size of the indenter should also be at least 8 times the sample size to ensure that the cartilage reacts as if it were part of an indefinite sample^22^. Using an indenter much smaller than the radius of the sample diameter eliminates any edge effects present in specimen creation. In addition, indentation avoids possible experimental errors caused by testing cartilage defects damaged by sample extraction. Indentation also does not involve deep sample preparation, such as confined compression, allowing small, thin pieces of cartilage to be tested^17^^,^^21^. Furthermore, the non-destructive method of indentation means that it has a potential application in the clinical setting as a diagnostic tool after validation and verification studies have been performed.

There are key assumptions with indentation that the user must ensure for appropriate results. A critical boundary condition in indentation loading requires constant contact between the indenter and the cartilage surface (*i.e.,* that the surface does not deform away from the indenter)^23^^,^^24^. Indentation loading also includes the assumed boundary condition that the contact between the cartilage surface and the indenter is non-destructive (*i.e.,* that the indenter is in contact with the surface but does not go through the surface; the cartilage surface should not fail under the indenter)^25^^-^^26^. Studies have shown that this boundary condition can be verified through use of India ink, which will stain damaged areas when applied to the cartilage surface^25^^,^^26^. A further boundary condition assumes that the indenter compresses the cartilage perpendicular to the surface of the sample. The perpendicular orientation of the compression is an important boundary condition because compressing at an angle, especially if using cyclic loading, may cause slippage, which may induce shearing components and change the mechanical loading. This condition may be ensured through careful test equipment set up.

After the summarized protocols have been optimized for the soft tissue of interest, it would be useful for researchers to look into dynamic testing of the tissue of interest. Appropriate cyclical loading of specimens should mimic normal physiological limits and behavior, such as mimicking walking or other repetitive movements^27^. In summary, this report demonstrates simple mechanical testing protocols to evaluate human tissues. Implementing these protocols will provide key information on the biomechanical characteristics of tissues, enabling tissue-engineered constructs to better mimic the native tissue.

## Disclosures

The authors have nothing to declare. 
